# A unique thymus-derived regulatory T cell subset associated with systemic lupus erythematosus

**DOI:** 10.1186/s13075-020-02183-2

**Published:** 2020-04-21

**Authors:** Hironari Hanaoka, Tetsuya Nishimoto, Yuka Okazaki, Tsutomu Takeuchi, Masataka Kuwana

**Affiliations:** 1 Division of Rheumatology, Department of Internal Medicine, Keio University School of Medicine, 35 Shinanomachi, Shinjuku-ku, Tokyo, 160-8582 Japan; 2 Department of Allergy and Rheumatology, Nippon Medical School Graduate School of Medicine, 1-1-5 Sendagi, Bunkyo-ku, Tokyo, 113-8603 Japan

**Keywords:** Systemic lupus erythematosus, Regulatory T cells, Th17, T cell plasticity

## Abstract

**Background:**

Foxp3 is a marker for regulatory T cells (Treg cells), but recent studies have shown the plasticity and heterogeneity of CD4^+^Foxp3^+^ T cells. This study aimed to examine the phenotype and function of circulating CD4^+^Foxp3^+^ T cells in patients with systemic lupus erythematosus (SLE).

**Methods:**

We enrolled 47 patients with SLE, 31 with organ-specific autoimmune diseases (15 with multiple sclerosis and 16 with primary immune thrombocytopenia), and 19 healthy subjects. Peripheral blood mononuclear cells were used to evaluate the proportion and phenotype of CD4^+^Foxp3^+^ cells using multicolor flow cytometry, the status of the Treg-specific demethylated region (TSDR) of the *foxp3* gene by methylation-specific polymerase chain reaction, and the immunoregulatory function of CD4^+^CD25^+^ cells by allogeneic mixed lymphocyte reaction. Immunohistochemistry of renal biopsy specimens obtained from 6 patients with lupus nephritis and 5 with IgA nephropathy was conducted to detect IL-17A-expressing CD4^+^Foxp3^+^ cells.

**Results:**

CD4^+^Foxp3^+^ T cells were increased in SLE patients compared with organ-specific autoimmune disease controls or healthy controls. Circulating CD4^+^Foxp3^+^ T cells were correlated with the disease activity of SLE. The increased CD4^+^Foxp3^+^ T cells in active SLE patients were mainly derived from thymus-derived Treg (tTreg) cells, as determined by a demethylated TSDR status, and represented a unique phenotype, upregulated expression of CD49d, CD161, and IL-17A, with immunosuppressive ability comparable to that of healthy controls. Finally, CD4^+^Foxp3^+^IL-17A^+^ cells were infiltrated into the renal biopsy specimens of patients with active lupus nephritis.

**Conclusions:**

A unique tTreg subset with dichotomic immunoregulatory and T helper 17 phenotypes is increased in the circulation of SLE patients and may be involved in the pathogenic process of SLE.

## Background

Forkhead box P3 (Foxp3)-positive regulatory T cells (Treg cells) are key mediators of peripheral self-tolerance that are able to actively suppress effector T cells, inhibit inflammation, and prevent autoimmunity [1, 2]. The expression of Foxp3, considered a master regulator of Treg cell development and function, is essential for the role of Treg cells in the maintenance of immune tolerance, but recent studies have shown plasticity and heterogeneity within CD4^+^Foxp3^+^ T cells, which include thymus-derived Treg (tTreg) cells, which are directly developed from CD4 single-positive cells in the thymus, peripherally derived Treg (pTreg) cells, which are differentiated from naïve CD4^+^ T cells in the periphery, and a subpopulation of activated effector T cells [3, 4]. Functionality of tTreg cells strongly depends on expression of Foxp3, but tTreg cells are not a homogeneous cell population and show diverse functional properties with various expression of surface markers and soluble mediators [5].

It is known that numeric abnormalities and functional impairment of Treg cells potentially contribute to the pathogenesis of various autoimmune diseases, including rheumatoid arthritis (RA) and organ-specific autoimmune diseases such as myasthenia gravis (MG), immune thrombocytopenia (ITP), and multiple sclerosis (MS) [6–11]. Furthermore, genetic mutations in the *foxp3* gene lead to functional impairment of Treg cells, resulting in the development of severe autoimmune and inflammatory conditions [12].

Systemic lupus erythematosus (SLE) is characterized by a breakdown of peripheral tolerance to a variety of self-antigens, followed by activation and expansion of autoreactive effector T and B cells, resulting in multiple organ damage through production of pathogenic autoantibodies and resultant immune complex deposition [13]. It has been shown that dysregulated adaptive and innate immune systems contribute to the pathophysiology of SLE [14, 15]. Since Treg cells play a major role in maintaining immune tolerance in the periphery, the numbers and function of CD4^+^Foxp3^+^ T cells in SLE patients have been extensively studied in recent years [16–23]. However, these studies have demonstrated quite contradictory results: some reported a reduced frequency and/or impaired regulatory function of circulating Foxp3^+^ Treg cells [19–21] in SLE patients in comparison to healthy controls, but others found an increased or comparable frequency of circulating Foxp3^+^ Treg cells [22, 23]. A recent meta-analysis revealed that the pooled percentage of CD4^+^Foxp3^+^ T cells in active SLE patients was found to be lower than that in controls, with great heterogeneity [24]. These discrepancies likely arise from the heterogeneity of CD4^+^Foxp3^+^ T cells and the difference in the combination of markers used in the flow cytometric analysis. Nevertheless, in this study, we investigated CD4^+^Foxp3^+^ T cell subsets associated with SLE by focusing on the heterogeneity of phenotypes and function of CD4^+^Foxp3^+^ T cells.

## Methods

### Patients and controls

This study used peripheral blood samples from 47 patients with SLE, who were consecutive patients visiting a rheumatology clinic at Keio University Hospital. All patients fulfilled the 1997 American College of Rheumatology revised criteria for the classification of SLE [25]. Patients taking > 20 mg of a prednisolone equivalent daily were excluded. Nineteen age/sex-matched healthy subjects were used as a control. In addition, 15 patients with MS and 16 with primary ITP were used as disease controls, since MS and ITP were shown to have dysregulated Treg/Th17 balance that potentially contributes to the pathogenesis [11]. All patients with MS or primary ITP satisfied the published criteria [26, 27].

We also used renal biopsy specimens of patients with lupus nephritis, independent of the analysis using peripheral blood samples. A reason for selecting kidney samples for the analysis was simply the availability of the affected organ samples obtained from SLE patients. Renal biopsy specimens were randomly selected from our renal biopsy bank: 6 samples of diffuse proliferative lupus nephritis classified as class IV-G (A/C) according to the International Society of Nephrology/Renal Pathology Society classification [28] and 5 samples of histologically confirmed IgA nephropathy. All samples were obtained after the subjects gave their written informed consent, as approved by the Institutional Review Board.

### Clinical characteristics

Through a retrospective chart review conducted at the same time as blood sampling or renal biopsy, demographic and clinical features, laboratory results, and treatment regimens were recorded in individual SLE patients. We also recorded the SLE disease activity index (SLEDAI) [29], 50% complement hemolytic activity (CH50) value, and the titers of serum anti-double-stranded DNA (dsDNA) antibodies, which were measured using a commercial enzyme-linked immunosorbent assay kit (MESACUP® DNA-II test, MBL, Nagoya, Japan) according to the manufacturer’s instructions. Active SLE was defined as not satisfying Lupus Low Disease Activity State [30], and mild, moderate, and severe disease activity were defined according to the recommendations [31].

### Cell preparation

Peripheral blood mononuclear cells (PBMCs) were isolated from heparinized venous blood using Lymphoprep (Fresenius Kabi AG, Bad Homburg, Germany) density gradient centrifugation. In some experiments, CD4^+^ cells, CD4^+^CD25^+^ cells, and CD4^+^CD25^−^ cells were isolated from PBMCs using magnetic cell sorting (MACS) column separation (Miltenyi Biotec, Bergisch Gladbach, Germany) according to the manufacturer’s protocol. In addition, a FACS Vantage flow cytometer (Becton Dickinson, Franklin Lakes, NJ, USA) was used to isolate CD4^+^Foxp3^+^ cells and CD4^+^Foxp3^−^ cells from PBMCs. The sorted fraction consistently contained > 90% of targeted cells as confirmed by flow cytometric analysis.

### Flow cytometry

For multicolor flow cytometric analysis, PBMCs were stained with the following fluorescence-conjugated monoclonal antibodies against CD4 (clone 13B8.2; Beckman Coulter Inc., Indianapolis, IN, USA), CD25 (clone M-A251; Becton Dickinson), CD49d (clone 9F10; Becton Dickinson), CD127 (clone HIL-7R-M21; Becton Dickinson), CD152 (clone L3D10; Biolegend, San Diego, CA, USA), and CD161 (clone HP-3G10; Biolegend). Cells were then fixed and permeabilized using the anti-human Foxp3 staining set (eBioscience, San Diego, SC, USA) followed by intracellular staining with monoclonal antibodies against Foxp3 (clone 236A/E7; eBioscience), IFN-γ (clone B27; Biolegend), IL-2 (clone MQ1–17H12; Biolegend), IL-17 (clone N49–653; Biolegend), and Helios (clone 22F6; Biolegend) according to the manufacturer’s instructions. The cells were analyzed on a FACS Calibur (Becton Dickinson) or FACS MoFlo (Beckman Coulter Inc.). In some experiments, the expression level of Foxp3 was evaluated using the mean fluorescent intensity (MFI).

### Analysis of the methylation status of the Treg-specific demethylated region (TSDR)

Demethylation of the TSDR within the promoter region of the *foxp3* gene is associated with stable expression of Foxp3 and is a unique property of tTreg cells [32, 33]. To evaluate the methylation status of the TSDR, methylation-specific polymerase chain reaction (PCR) was used as previously described [34]. Briefly, genomic DNA was extracted from isolated CD4^+^Foxp3^+^ and CD4^+^Foxp3^−^ cells with the QIAamp DNA blood mini kit (Qiagen Inc., Valencia, CA, USA) and then subjected to bisulfite conversion with the Cell-to-CpG™ Bisulfite Conversion kit (Applied Biosystems, Foster City, CA, USA) according to the manufacturer’s instructions. Bisulfite-treated genomic DNA was subjected to real-time PCR to analyze the methylation status of the TSDR using methylated DNA or demethylated DNA-specific probes and primers [34]. The proportion of cells with a demethylated TSDR was calculated using the following formula: [number of demethylated TSDR sequences] / [sum of number of demethylated and methylated TSDR sequences] × [number of X chromosomes per cell]. All analyses were conducted in duplicate.

### Analysis of immunosuppressive function

The immunosuppressive capacity of CD4^+^CD25^+^ T cells was examined using allogeneic mixed lymphocyte reaction (MLR) as previously described [35]. Briefly, CD4^+^CD25^−^ cells (10^4^), which were used as effector T cells, were labeled with 1 μM carboxyfluorescein succinimidyl ester (CFSE) (Invitrogen) and cultured with irradiated (60 Gy) allogeneic PBMCs (10^5^) as feeder cells in RPMI 1640 medium supplemented with 10% fetal bovine serum, 100 IU/mL penicillin, and 100 mg/mL streptomycin in the presence or absence of CD4^+^CD25^+^ T cells (10^4^), which were used as Treg cells. Next, 96-well round-bottom plates were used after pretreatment with 0.5 μg/mL anti-CD3 monoclonal antibody (clone OKT3; Becton Dickinson). Phytohemagglutinin (PHA) was used as a control for T cell proliferation. After 5 days of culture, the proliferation of CFSE-labeled cells was assessed by flow cytometry. Immunosuppressive activity was calculated based on the following formula: (1-[proportion of proliferating cells in the culture of effector T cells and Treg cells] / [proportion of proliferating cells in the culture of effector T cells]) × 100 (%).

### Immunohistochemistry

Infiltration of IL-17-expressing CD4^+^Foxp3^+^ T cells was evaluated by immunohistochemistry of renal biopsy specimens as previously described with some modifications [34]. Briefly, paraffin-embedded sections (5-μm thickness) of renal biopsy samples were boiled in 10 mM citrate buffer (pH 6.0) and blocked with 5% bovine serum albumin. Subsequently, the sections were incubated with anti-CD4 monoclonal antibody (1:100, clone mAbcam51312, Abcam), anti-Foxp3 monoclonal antibody (1:50, clone mAbcam22510, Abcam), and anti-IL-17A rabbit polyclonal antibody (1:100, Abcam), followed by incubation with fluorescence-conjugated secondary antibodies (Molecular Probes). Images were taken using a Fluoview FV1000 confocal laser fluorescence microscope (Olympus, Tokyo, Japan). The proportions of Foxp3^+^ cells in CD4^+^IL-17A^+^ cells were determined by counting at least 50 cells of the cells positive for IL-17A.

### Statistical analysis

Continuous data are shown as box-whisker plots or the mean ± standard deviation. The box plots contain a central rectangle (box) with lines (whiskers) that extend from both ends and provide information about the smallest value, first quartile (Q1), median, third quartile (Q3), and largest value. All statistical analyses were performed using IBM SPSS statistics 22 (IBM Corporation, Armonk, NY, USA). Comparisons between two groups were tested for statistical significance using the nonparametric Mann-Whitney *U* test. Comparisons among 3 or more groups were performed using ANOVA followed by Bonferroni’s test. The correlation coefficient was obtained by Spearman’s correlation analysis. *P* < 0.05 was considered significant.

## Results

### Demographic and clinical characteristics of SLE patients used for peripheral blood analysis

The age at enrollment was 42.4 ± 14.8, 44.9 ± 9.1, 51.5 ± 16.7, and 42.2 ± 10.8 years, and the proportion of females was 70%, 60%, 50%, and 68% in patients with SLE, MS, primary ITP, and healthy controls, respectively. There was no difference in age and sex distribution among groups, while age tended to be older in patients with primary ITP than that in the other groups. The clinical characteristics of the SLE patients used for analysis are shown in Table 1. The disease activity, distribution of organ involvement, and treatment were variable among patients: this was expected because consecutive patients in routine clinical practice were enrolled. Nevertheless, it is apparent that the enrolled subjects included the patients with apparent disease activity with elevated anti-dsDNA antibody levels and increased SLEDAI at blood draw. Lupus nephritis was manifested in 42.5% of the patients. SLE patients took a mean of 10.9 ± 9.6 mg/day of prednisolone with a variety of immunosuppressive agents. No patients took hydroxychloroquine.

**Table 1:** Characteristics of SLE patients used for analysis

Number	47
Age, year	42.4 ± 14.8
Female, number (%)	33 (70)
SLEDAI	5.0 ± 4.8
CH50, U/mL	31.7 ± 12.0
Anti-dsDNA antibody, U/mL	105.0 ± 134.2
CRP, mg/dL	0.4 ± 0.9
Organ manifestations	
Skin, number (%)	11 (23.4)
Arthritis, number (%)	10 (21.3)
Cytopenia, number (%)	18 (38.3)
Lupus nephritis, number (%)	20 (42.5)
Serositis, number (%)	7 (14.9)
Immunosuppressive therapy	
Prednisolone, mg/day	10.9 ± 9.6
Azathioprine, number (%)	10 (21.3)
Methotrexate, number (%)	9 (19.1)
Tacrolimus, number (%)	12 (25.6)
Cyclosporine, number (%)	4 (8.5)
Hydroxychloroquine, number (%)	0 (0)

*SLE* systemic lupus erythematosus, *SLEDAI* systemic lupus erythematosus disease activity index; dsDNA, double-stranded DNA, *CH50* 50% complement hemolytic activity

### Increased proportion of CD4^+^Foxp3^+^ T cells in the circulation of active SLE patients

**Fig. 1:**
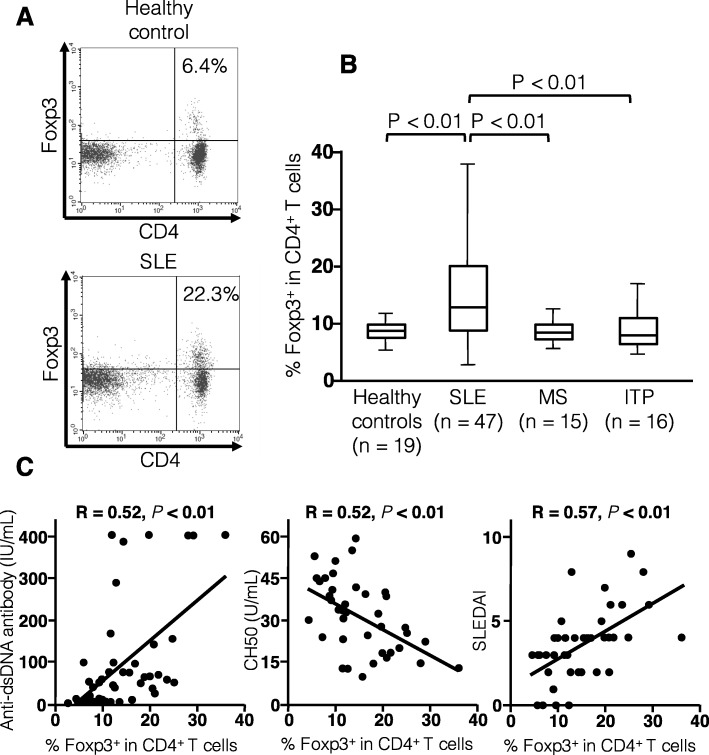
Proportion of circulating CD4^+^Foxp3^+^ T cells in SLE patients and controls. **a** Representative dot plot analysis in a healthy donor and a patient with SLE. **b** Proportion of CD4^+^Foxp3^+^ T cells in healthy controls (*n* = 19), SLE patients (*n* = 47), MS patients (*n* = 15) and primary ITP patients (*n* = 16). **c** Correlations between the proportion of CD4^+^Foxp3^+^ T cells and anti-double-stranded DNA (dsDNA) antibody levels, CH50 values, and SLE disease activity index (SLEDAI) scores in 47 patients with SLE

We first examined the frequencies of Foxp3^+^ cells in circulating CD4^+^ T cells using flow cytometry (Fig. 1). The proportion of CD4^+^Foxp3^+^ T cells was increased in SLE patients compared to healthy controls, patients with MS, or those with primary ITP (*P* < 0.01 in all comparisons). In SLE patients, the proportion of CD4^+^Foxp3^+^ T cells was positively correlated with anti-dsDNA antibody titer (r = 0.57, *P* < 0.01) and SLEDAI (r = 0.52, *P* < 0.01) and negatively correlated with CH50 levels (r = 0.52, P < 0.01).

**Fig. 2:**
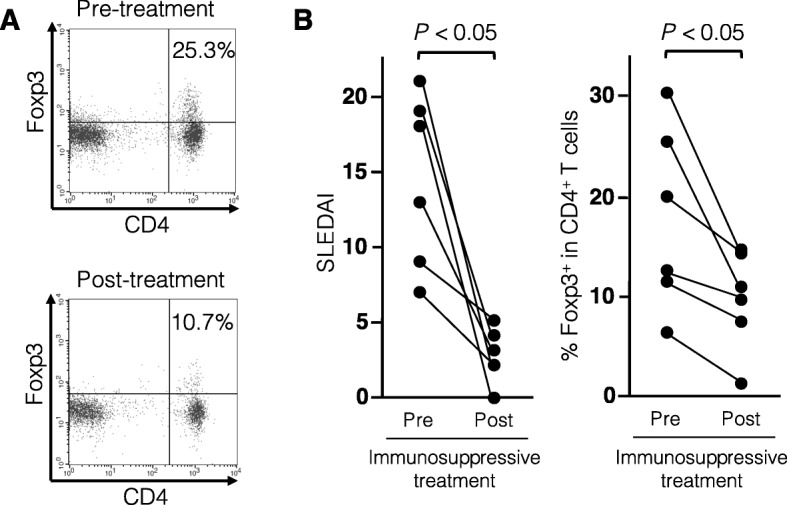
Serial analysis of the proportion of circulating CD4^+^Foxp3^+^ T cells in patients with active SLE. **a** Representative dot plot analysis in a patient with SLE before and after immunosuppressive treatment. **b** Changes in SLEDAI score and the proportion of CD4^+^Foxp3^+^ T cells before and after treatment in 6 patients with SLE

Peripheral blood samples of 6 patients with moderate or high disease activity at enrollment (4 with active nephritis with histologic confirmation of class IV-G, and 2 with pleuritis, skin rash, and cytopenia) were available after the remission induction treatment. The treatment regimens used for all patients were high-dose corticosteroids combined with an immunosuppressant (4 with intravenous cyclophosphamide, and 2 with tacrolimus). In 6 SLE patients, the proportion of CD4^+^Foxp3^+^ T cells was significantly decreased after immunosuppressive treatment, which resulted in a reduction in SLEDAI (*P* < 0.01) (Fig. 2). These results together suggest that the increased proportion of CD4^+^Foxp3^+^ T cells in circulation is specific to SLE and correlates with disease activity. Therefore, samples obtained from patients with active SLE were used in the following experiments.

### Origin of CD4^+^Foxp3^+^ T cells increased in the circulation of active SLE patients

**Fig. 3:**
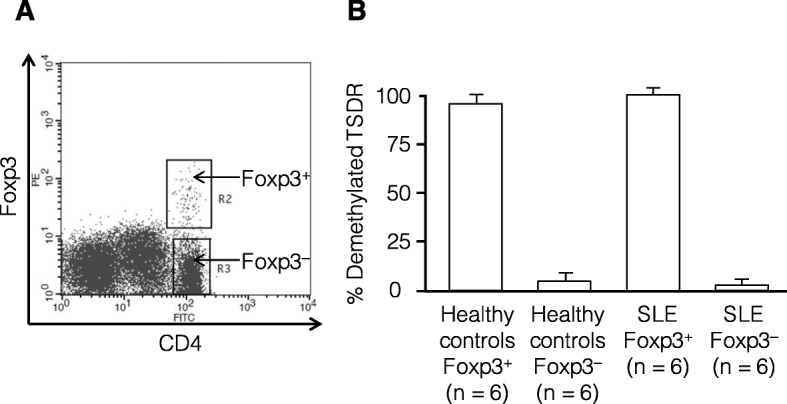
Methylation status at the TSDR of the *foxp3* gene in CD4^+^Foxp3^+^ T cells in active SLE patients and healthy controls. **a** A representative dot plot of the target cell populations sorted using a FACS Vantage flow cytometer: CD4^+^Foxp3^+^ cells and CD4^+^Foxp3^−^ cells. **b** The mean proportion of demethylated TSDRs in isolated CD4^+^Foxp3^+^ cells and CD4^+^Foxp3^−^ cells in healthy controls (*n* = 6) and active SLE patients (n = 6)

To assess whether the increased CD4^+^Foxp3^+^ T cells in the circulation of SLE patients were derived from the thymus, the methylation status at the TSDR of the *foxp3* gene was examined by methylation-specific PCR. To examine whether the TSDRs of CD4^+^Foxp3^+^ T cells in SLE patients were demethylated, CD4^+^Foxp3^+^ and CD4^+^Foxp3^−^ cells were individually isolated from the PBMCs of 6 active SLE patients and 6 heathy controls using flow cytometer-based sorting and subjected to methylation-specific PCR (Fig. 3a and b). The TSDR of the *foxp3* gene of CD4^+^Foxp3^+^ T cells was completely demethylated in both SLE patients and healthy controls. This finding indicates that the increased CD4^+^Foxp3^+^ T cells in the circulation of active SLE patients originated mainly from tTreg cells.

### Altered phenotype of CD4^+^Foxp3^+^ T cells in the circulation of active SLE patients

**Fig. 4:**
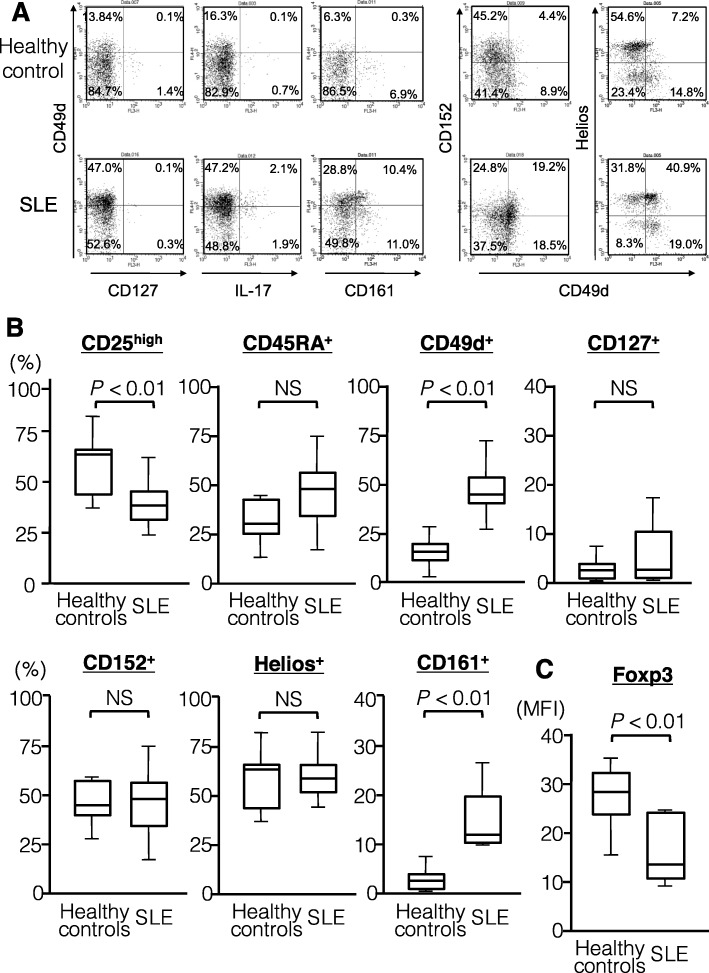
Phenotypic properties of CD4^+^Foxp3^+^ T cell subsets in active SLE patients and healthy controls. **a** Representative dot-plot analysis in a healthy donor and a patient with active SLE. **b** Proportions of the cells with Treg and Th17 markers, including CD25high, CD45RA, CD49d, CD127, CD152, Helios, and CD161, in CD4^+^Foxp3^+^ T cells from healthy controls (n = 15) and active SLE patients (n = 16). **c** The expression level of Foxp3 in CD4^+^Foxp3^+^ T cells from healthy controls (n = 15) and active SLE patients (n = 16). NS = not significant

**Fig. 5:**
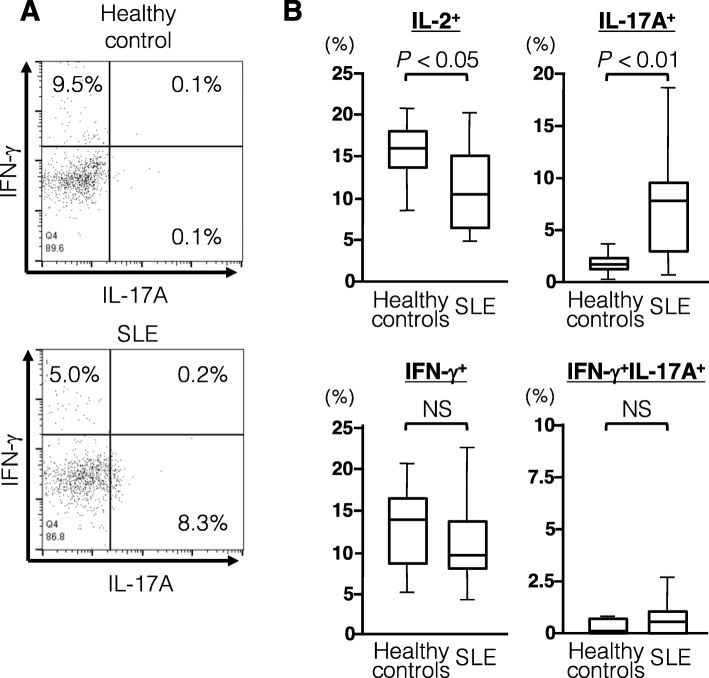
Cytokine expression profiles of CD4^+^Foxp3^+^ T cells in active SLE patients and healthy controls. **a** Representative dot plot analysis for the expression of IFN-γ and IL-17 on CD4^+^Foxp3^+^ T cells from a healthy donor and a patient with active SLE. **b** Proportions of T cells expressing IL-2, IL-17, IFN-γ, or both IFN-γ and IL-17 in CD4^+^Foxp3^+^ T cells from healthy controls (*n* = 11) and active SLE patients (n = 11). NS = not significant

We next evaluated the phenotypes of CD4^+^Foxp3^+^ T cells increased in circulation of active SLE patients using multicolor flow cytometry. We examined the expression of CD25, CD45RA, CD127, CD49d, CD161, CD152, and Helios as well as cytokines, including IL-2, IL-17, and IFN-γ, on gated CD4^+^Foxp3^+^ T cells in active SLE patients and healthy controls (Fig. 4). In CD4^+^Foxp3^+^ T cells from SLE patients, CD25^high^ cells were less frequent but CD49d^+^ cells were more frequent than the respective abundances in CD4^+^Foxp3^+^ T cells from heathy controls (*P* < 0.01 for both comparisons). There was no difference in the proportions of CD45RA^+^, CD127^+^, CD152^+^, and Helios^+^ cells between SLE and healthy controls, whereas the proportion of CD161^+^ cells was markedly increased in active SLE patients versus healthy controls (*P* < 0.01). Finally, the expression level of Foxp3 within CD4^+^Foxp3^+^ T cells was significantly lower in active SLE patients than in healthy controls (*P* < 0.01). In terms of cytokine expression profiles, lower IL-2 and higher IL-17A were observed in the CD4^+^Foxp3^+^ T cells from active SLE patients compared with the respective levels in those from healthy controls (*P* < 0.05 for both comparisons), whereas IFN-γ expression was comparable (Fig. 5). These findings together suggest that the increased CD4^+^Foxp3^+^ T cells in the circulation of active SLE patients consist mainly of Foxp3^low^CD45RA^+^CD25^low^ cells with higher expression of CD49d, CD161, and IL-17A but comparable expression of CD152 and Helios.

### Immunosuppressive function of CD4^+^CD25^+^ T cells in the circulation of active SLE patients

**Fig. 6:**
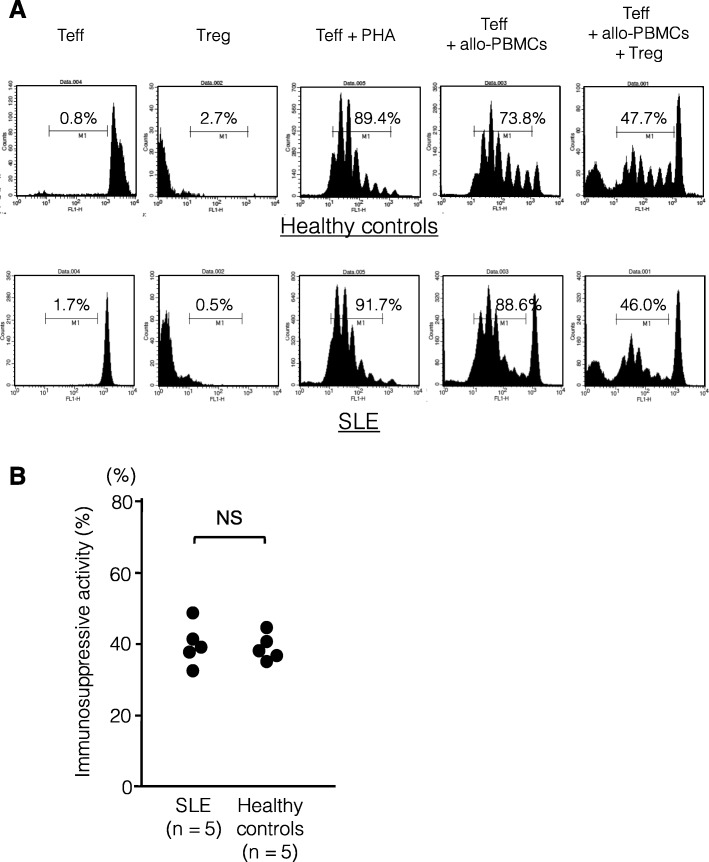
In vitro immunosuppressive potency of CD4^+^CD25^+^ T cells assessed by allogeneic mixed lymphocyte reaction in active SLE patients and healthy controls. Allogeneic peripheral blood mononuclear cells (allo-PBMCs) were cocultured with flow cytometer-sorted CD4^+^CD25^+^ cells, which were regarded as T regulatory cells (Treg cells), and/or CD4^+^CD25^−^ cells, which were regarded as effector T cells (Teff cells). Cell proliferation was quantitatively assessed using CSFE labeling. PHA was used as a control for cell proliferation. **a** Representative histograms for cell proliferation in a healthy donor and a patient with SLE. **b** Immunosuppressive activity of CD4^+^CD25^+^ T cells in active SLE patients (*n* = 5) and healthy controls (n = 5). NS = not significant

The in vitro immunosuppressive potency of CD4^+^CD25^+^ T cells was assessed using cultures of allogeneic MLR (Fig. 6). For this purpose, we isolated CD4^+^CD25^+^ and CD4^+^CD25^−^ cells from PBMCs of active SLE patients and healthy controls as Treg cells and effector T cells, respectively. The isolated CD4^+^CD25^+^ cells constantly contained > 90% CD4^+^Foxp3^+^ T cells. In representative assays using samples from an active SLE patient and a healthy control, the proliferation of effector T cells induced by allogeneic PBMCs was similarly suppressed by coculture with CD4^+^CD25^+^ cells. There was no difference in the immunosuppressive activity of CD4^+^CD25^+^ T cells between active SLE patients and healthy controls.

### Foxp3 expression in CD4^+^IL-17A^+^ cells infiltrated into renal biopsy specimens

**Fig. 7:**
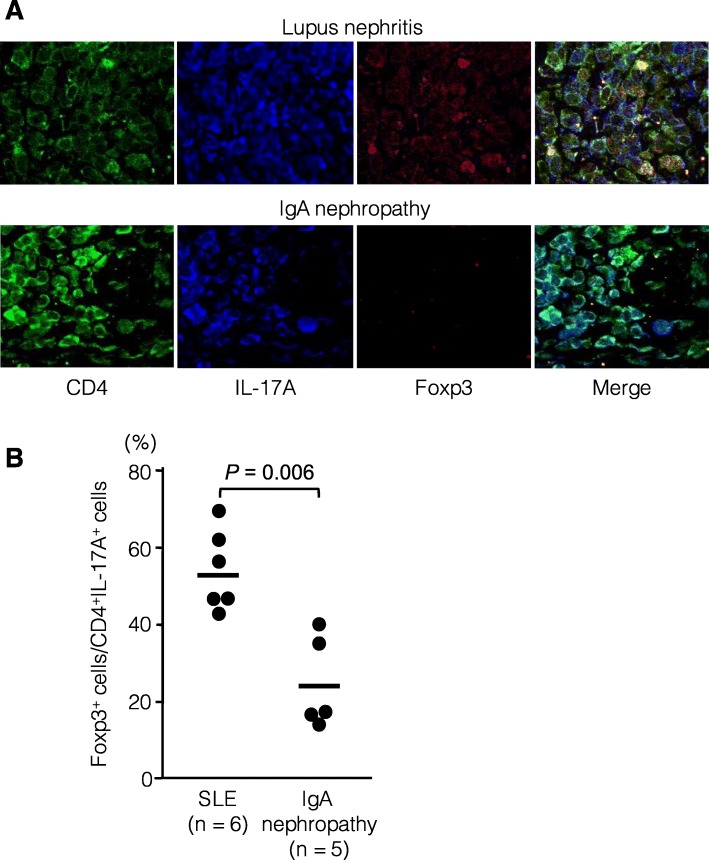
Detection of infiltrating CD4^+^Foxp3^+^IL-17^+^ T cells in renal biopsy samples from patients with lupus nephritis and IgA nephropathy. **a** Representative images of immunohistochemical staining for CD4 (cell surface), IL-17A (cytoplasm), and Foxp3 (cytoplasm and nucleus). Magnification = × 400. **b** Proportions of Foxp3^+^ T cells in CD4^+^IL-17^+^ T cells infiltrated in renal specimens from patients with lupus nephritis (n = 6) and those with IgA nephropathy (n = 5)

We further examined the potential recruitment of CD4^+^Foxp3^+^IL-17A^+^ T cells in the affected organs of SLE patients. For this purpose, renal biopsy specimens from patients with active lupus nephritis and those with IgA nephropathy were randomly selected from our renal biopsy bank, and subjected to immunohistochemistry (Fig. 7). Clinical characteristics of the 6 patients with class IV-G (A/C) lupus nephritis at renal biopsy included proteinuria of 4.7 ± 0.7 g/day, glomerular filtration rate of 82.4 ± 0.7 mL/min/1.73 m^2^, SLEDAI of 14.3 ± 1.9, anti-dsDNA antibody levels of 148.7 ± 28.9 U/mL, and CH50 of 18.7 ± 0.9 U/mL, on treatment with 13.0 ± 4.7 mg/day of prednisolone without immunosuppressant. CD4^+^IL-17A^+^ cells were detected mainly in the interstitial lesions of kidneys from both patients with lupus nephritis and those with IgA nephropathy, but the majority of the CD4^+^IL-17A^+^ cells also showed nuclear/cytoplasmic expression of Foxp3 in patients with lupus nephritis. The proportion of Foxp3^+^ cells in CD4^+^IL-17A^+^ cells was significantly greater in patients with lupus than in those with IgA nephropathy. Correlations of proportions of CD4^+^Foxp3^+^IL-17A^+^ T cells in the kidney and peripheral blood were not evaluable because peripheral blood samples of the patients at renal biopsy were not available.

## Discussion

Our results demonstrated that circulating CD4^+^Foxp3^+^ T cells were increased in SLE patients in association with disease activity. Most of the increased CD4^+^Foxp3^+^ T cells in the peripheral blood of active SLE patients originated from tTreg cells, as determined by the completely demethylated status at the TSDR of the *foxp3* gene, and were consistent with naïve or resting Treg cells, as reported by Miyara et al. [36]. This tTreg subset represented a unique phenotype, upregulated expression of CD49d, expression of the authentic Treg markers Helios and CD152, expression of the T helper 17 (Th17) marker CD161, and expression of IL-17A, and the cell subset showed immunosuppressive ability. Finally, IL-17A-expressing CD4^+^Foxp3^+^ T cells were also detected in renal biopsy specimens of patients with active lupus nephritis. Since depressed Treg cell function and elevated Th17 response are critical to the pathogenesis of SLE [37], these results together indicated that a unique tTreg subset acquiring both immunoregulatory and Th17 phenotypes might be involved in the pathogenic process of SLE by expanding in circulation and infiltrating into the affected tissue. It is interesting to note that this unique tTreg subset has two different aspects in terms of SLE pathogenesis, including immunosuppressive and proinflammatory properties, although our study failed to draw any conclusion whether they are harmful or protective.

Our findings were principally consistent with previous studies showing increased tTreg cells with a demethylated TSDR of the *foxp3* gene locus in the peripheral blood of SLE patients [36, 38–41]. Although the markers used to evaluate CD4^+^Foxp3^+^ T cell subsets were variable among previous studies, resulting in difficulty in comparing the findings described in individual reports, the CD4^+^Foxp3^+^ T cell subsets reported to be increased in SLE patients included Foxp3^low^CD45RA^+^CD25^low^ cells (fraction I) [36], Helios^+^ T cells [40, 41], CD161^+^ T cells [42], and IL-17-producing T cells [39], which were consistent with the unique tTreg subset identified in this study.

It has been recently reported that Treg cells become unstable under certain inflammatory and/or pathologic conditions and adopt characteristics of effector CD4^+^ T cells [3, 11, 43]. In particular, Foxp3^+^ Treg cells are able to differentiate into IL-17-producing Th17-like cells upon receiving external signals. The first study in mice showed that transforming growth factor-β induced differentiation of CD4^+^Foxp3^+^RORγt^+^ T cells with the ability to produce IL-17 [44], but subsequent studies using human peripheral blood and secondary lymphoid tissue identified similar CD4^+^Foxp3^+^ T cell subsets producing IL-17 and inhibiting the proliferation of CD4^+^ responder T cells [42, 45]. These CD4^+^Foxp3^+^ T cell subsets with both Treg and effector T cell properties were characterized by a demethylated status of TSDRs of the *foxp* gene locus, immunosuppressive function, and production of pro-inflammatory cytokines, including IL-17 [46]. There is accumulating evidence showing the plasticity of Treg cells and their role in autoimmunity, and these bipotential Treg cell subsets have been shown to be involved in pathogenic processes in a variety of autoimmune mouse models, such as arthritis, nephritis, and colitis [43, 47, 48]. In addition, CD4^+^ T cells expressing both Foxp3 and IL-17 have been shown to be increased in the peripheral blood of patients with various autoimmune diseases, including systemic sclerosis, RA, and Crohn’s disease [35, 47, 49, 50], although the analysis of TSDRs of the *foxp3* gene locus was not conducted in some studies, resulting in difficulty in discriminating between CD4^+^Foxp3^+^ T cell subsets originating from tTreg cells, pTreg cells, and effector T cells. Nevertheless, Komatsu et al. [47] reported infiltration of CD4^+^Foxp3^+^IL-17A^+^ T cells in the inflamed synovium of RA patients, similar to our findings observed in the renal tissue of SLE patients, suggesting the pathological importance of plastic tTreg cells in systemic autoimmune diseases. We used renal biopsy specimens as one of the affected organs of SLE due to sample availability, it is interesting to examine whether CD4^+^Foxp3^+^IL-17A^+^ T cells are recruited to other manifested organs of SLE.

One of the notable features of IL-17-expressing tTreg cells detected in active SLE patients is high expression of CD49d, also called α4 integrin, which forms a complex with β1 or β7 integrin. There is little information on the function of CD49d^+^ Treg cells, but one study reported a lower immunosuppressive capacity of CD4^+^CD45RA^−^ effector T cells than CD49d^−^ Treg cells [51]. CD49d is involved in the migration of activated effector CD4^+^ T cells, including Th1 cells and Th17 cells, into inflamed tissue, such as the central nervous system, intestine, and kidneys [45, 52, 53]. In a rat autoimmune experimental nephritis model, reduction in the CD49d expression on lymphocytes and the resultant inhibition of their adhesion led to complete resolution of nephritis [54]. Therefore, it is possible that CD49d expressed on Th17-like tTreg cells contributes to preferential infiltration of potential pathogenic T cells into affected tissue.

It has been shown that the fate of tTreg cells towards differentiation into inflammatory T cells is mediated in a signal transducer and activator of transcription 3 (STAT3)-dependent manner [55, 56]. In this regard, Th17-like cells can be induced from tTreg cells in vitro by stimulation with a combination of high-mobility group box-1 protein (HMGB-1) and IL-6 through enhanced STAT3 signaling [39]. A critical role of IL-6 in inducing Th17 cells has been well recognized [57]. HMGB-1 is a damage-associated molecular pattern (DAMP) and a ligand of Toll-like receptor (TLR)2 and TLR4 [58]. It is interesting to note that HMGB-1 is released from apoptotic cells, which are abundant in the circulation and lymphoid tissues of patients with active SLE [59]. On the other hand, IL-21 stimulated mammalian target of rapamycin (mTOR) complexes 1 and 2, abrogated the autophagy, differentiation, and function of Treg cells in a STAT3-dependent manner, and drove expansion of Th17-like cells in SLE patients [60, 61]. Upregulation of IL-21 was also reported in patients with active SLE [62]. Finally, it is known that IL-2 regulates the homeostasis of CD4^+^ T cells, and its deficiency may lead to the instability of Treg cells in patients with SLE [63, 64]. Further investigations of intracellular mechanisms regulating differentiation of tTreg cells into inflammatory T cells in SLE patients are necessary to provide insights into pathogenesis of immune dysregulation observed in SLE patients.

In mice, IL-2 enhances Treg cell development and survival and suppresses the differentiation of follicular helper T cells and Th17 subsets [65]. A recent study evaluating the efficacy of low-dose IL-2 treatment in patients with SLE found an increase in the number of Treg cells and suppression of follicular helper T cells and Th17 cell numbers in peripheral blood, accompanied by a marked reduction in disease activity [66]. A recent intriguing report described the first case of a patient with SLE treated with autologous adoptive Treg cell therapy, which led to increased activated Treg cells in the inflamed skin, with a marked attenuation of the IFN-γ pathway and a reciprocal augmentation of the IL-17 pathway [67]. This phenomenon was more pronounced in skin than in peripheral blood and was validated in a mouse model undergoing Treg cell adoptive transfer. These findings suggest that dysregulation of immune and inflammatory systems in SLE patients plays a role in converting tTreg cells into Th17-like cells.

## Conclusions

We have demonstrated that a dichotomic tTreg subset with both immunoregulatory and Th17 phenotypes is increased in the circulation of SLE patients. This tTreg subset might be involved in the pathogenic process of SLE by infiltrating into the effected tissue, although whether this subset is harmful or protective remains unclear. Further studies investigating the roles of this tTreg subset in the pathogenic process of SLE and the mechanisms underlying its differentiation are useful for understanding the pathogenesis of SLE and developing potential biomarkers and therapeutic targets.

## Data Availability

The datasets analyzed during the current study are available from the corresponding author upon reasonable request.

## Abbreviations

Foxp3: Forkhead box P3
Treg cells: regulatory T cells
tTreg: thymus-derived Treg
pTreg: peripherally derived Treg
MG: myasthenia gravis
ITP: immune thrombocytopenia
MS: multiple sclerosis
SLE: systemic lupus erythematosus
SLEDAI: SLE disease activity index
CH50: 50% complement hemolytic activity
PBMCs: peripheral blood mononuclear cells
MACS: magnetic-activated cell sorting
TSDR: Treg-specific demethylated region
MLR: mixed lymphocyte reaction
CFSE: carboxyfluorescein succinimidyl ester
PHA: phytohemagglutinin
Th17: T helper 17
STAT3: signal transducer and activator of transcription 3
HMGB-1: high-mobility group box-1 protein
DAMPs: damage-associated molecular patterns
TLR: Toll-like receptor
mTOR: mammalian target of rapamycin

## References

[1] Sakaguchi S, Miyara M, Costantino CM, Hafler DA (2010). FOXP3+ regulatory T cells in the human immune system. Nat Rev Immunol.

[2] Caramalho I, Nunes-Cabaco H, Foxall RB, Sousa AE (2015). Regulatory T-cell development in the human thymus. Front Immunol.

[3] Sakaguchi S, Vignali DA, Rudensky AY, Niec RE, Waldmann H (2013). The plasticity and stability of regulatory T cells. Nat Rev Immunol..

[4] Morikawa H, Sakaguchi S (2014). Genetic and epigenetic basis of Treg cell development and function: from a FoxP3-centered view to an epigenome-defined view of natural Treg cells. Immunol Rev.

[5] Schmetterer KG, Neunkirchner A, Pickl WF (2012). Naturally occurring regulatory T cells: markers, mechanisms, and manipulation. FASEB J.

[6] Nishimoto T, Kuwana M (2013). CD4+CD25+Foxp3+ regulatory T cells in the pathophysiology of immune thrombocytopenia. Semin Hematol.

[7] Dhaeze T, Stinissen P, Liston A, Hellings N (2015). Humoral autoimmunity: a failure of regulatory T cells?. Autoimmun Rev.

[8] Shevach EM (2000). Regulatory T cells in autoimmmunity. Annu Rev Immunol.

[9] Miyara M, Gorochov G, Ehrenstein M, Musset L, Sakaguchi S, Amoura Z (2011). Human FoxP3+ regulatory T cells in systemic autoimmune diseases. Autoimmun Rev.

[10] Gertel-Lapter S, Mizrachi K, Berrih-Aknin S, Fuchs S, Souroujon MC (2013). Impairment of regulatory T cells in myasthenia gravis: studies in an experimental model. Autoimmun Rev.

[11] Noack M, Miossec P (2014). Th17 and regulatory T cell balance in autoimmune and inflammatory diseases. Autoimmun Rev.

[12] Nie J, Li YY, Zheng SG, Tsun A, Li B (2015). FOXP3(+) Treg cells and gender bias in autoimmune diseases. Front Immunol.

[13] Mirabelli G, Cannarile F, Bruni C, Vagelli R, De Luca R, Carli L (2015). One year in review 2015: systemic lupus erythematosus. Clin Exp Rheumatol.

[14] Mohan C, Putterman C (2015). Genetics and pathogenesis of systemic lupus erythematosus and lupus nephritis. Nat Rev Nephrol.

[15] Konya C, Paz Z, Tsokos GC (2014). The role of T cells in systemic lupus erythematosus: an update. Curr Opin Rheumatol.

[16] Ohl K, Tenbrock K (2015). Regulatory T cells in systemic lupus erythematosus. Eur J Immunol.

[17] Brusko TM, Putnam AL, Bluestone JA (2008). Human regulatory T cells: role in autoimmune disease and therapeutic opportunities. Immunol Rev.

[18] Scheinecker C, Bonelli M, Smolen JS (2010). Pathogenetic aspects of systemic lupus erythematosus with an emphasis on regulatory T cells. J Autoimmun.

[19] Crispin JC, Martinez A, Alcocer-Varela J (2003). Quantification of regulatory T cells in patients with systemic lupus erythematosus. J Autoimmun.

[20] Lyssuk EY, Torgashina AV, Soloviev SK, Nassonov EL, Bykovskaia SN (2007). Reduced number and function of CD4+CD25highFoxP3+ regulatory T cells in patients with systemic lupus erythematosus. Adv Exp Med Biol.

[21] Liu MF, Wang CR, Fung LL, Wu CR (2004). Decreased CD4+CD25+ T cells in peripheral blood of patients with systemic lupus erythematosus. Scand J Immunol.

[22] Alvarado-Sanchez B, Hernandez-Castro B, Portales-Perez D, Baranda L, Layseca-Espinosa E, Abud-Mendoza C (2006). Regulatory T cells in patients with systemic lupus erythematosus. J Autoimmun.

[23] Vargas-Rojas MI, Crispin JC, Richaud-Patin Y, Alcocer-Varela J (2008). Quantitative and qualitative normal regulatory T cells are not capable of inducing suppression in SLE patients due to T-cell resistance. Lupus..

[24] Li W, Deng C, Yang H, Wang G (2019). The regulatory T cell in active systemic lupus erythematosus patients: a systemic review and meta-analysis. Front Immunol.

[25] Hochberg MC (1997). Updating the American college of rheumatology revised criteria for the classification of systemic lupus erythematosus. Arthritis Rheum.

[26] Thompson AJ, Banwell BL, Barkhof F, Carroll WM, Coetzee T, Comi G (2018). Diagnosis of multiple sclerosis: 2017 revisions of the McDonald criteria. Lancet Neurol.

[27] George JN, Woolf SH, Raskob GE, Wasser JS, Aledort LM, Ballem PJ (1996). Idiopathic thrombocytopenic purpura: a practice guideline developed by explicit methods for the American Society of Hematology. Blood..

[28] Weening JJ, D'Agati VD, Schwartz MM, Seshan SV, Alpers CE, Appel GB (2004). The classification of glomerulonephritis in systemic lupus erythematosus revisited. J Am Soc Nephrol.

[29] Bombardier C, Gladman DD, Urowitz MB, Caron D, Chang CH (1992). Derivation of the SLEDAI. A disease activity index for lupus patients. The committee on prognosis studies in SLE. Arthritis Rheum.

[30] Franklyn K, Lau CS, Navarra SV, Louthrenoo W, Lateef A, Hamijoyo L (2016). Definition and initial validation of a lupus low disease activity state (LLDAS). Ann Rheum Dis.

[31] Fanouriakis A, Kostopoulou M, Alunno A, Aringer M, Bajema I, Boletis JN (2019). 2019 update of the EULAR recommendations for the management of systemic lupus erythematosus. Ann Rheum Dis.

[32] Lal G, Bromberg JS (2009). Epigenetic mechanisms of regulation of Foxp3 expression. Blood..

[33] Huehn J, Beyer M (2015). Epigenetic and transcriptional control of Foxp3+ regulatory T cells. Semin Immunol.

[34] de Vries IJ, Castelli C, Huygens C, Jacobs JF, Stockis J, Schuler-Thurner B (2011). Frequency of circulating Tregs with demethylated FOXP3 intron 1 in melanoma patients receiving tumor vaccines and potentially Treg-depleting agents. Clin Cancer Res.

[35] Liu X, Gao N, Li M, Xu D, Hou Y, Wang Q (2013). Elevated levels of CD4(+)CD25(+)FoxP3(+) T cells in systemic sclerosis patients contribute to the secretion of IL-17 and immunosuppression dysfunction. PLoS One.

[36] Miyara M, Yoshioka Y, Kitoh A, Shima T, Wing K, Niwa A (2009). Functional delineation and differentiation dynamics of human CD4+ T cells expressing the FoxP3 transcription factor. Immunity..

[37] Yang J, Chu Y, Yang X, Gao D, Zhu L, Yang X (2009). Th17 and natural Treg cell population dynamics in systemic lupus erythematosus. Arthritis Rheum.

[38] Bonelli M, Savitskaya A, Steiner CW, Rath E, Smolen JS, Scheinecker C (2009). Phenotypic and functional analysis of CD4+ CD25- Foxp3+ T cells in patients with systemic lupus erythematosus. J Immunol.

[39] Jiang C, Wang H, Xue M, Lin L, Wang J, Cai G (2019). Reprograming of peripheral Foxp3+ regulatory T cell towards Th17-like cell in patients with active systemic lupus erythematosus. Clin Immunol.

[40] Alexander T, Sattler A, Templin L, Kohler S, Gross C, Meisel A (2013). Foxp3+ Helios+ regulatory T cells are expanded in active systemic lupus erythematosus. Ann Rheum Dis.

[41] Golding A, Hasni S, Illei G, Shevach EM (2013). The percentage of FoxP3+Helios+ Treg cells correlates positively with disease activity in systemic lupus erythematosus. Arthritis Rheum.

[42] Pesenacker AM, Bending D, Ursu S, Wu Q, Nistala K, Wedderburn LR (2013). CD161 defines the subset of FoxP3+ T cells capable of producing proinflammatory cytokines. Blood..

[43] Kleinewietfeld M, Hafler DA (2013). The plasticity of human Treg and Th17 cells and its role in autoimmunity. Semin Immunol.

[44] Zhou L, Lopes JE, Chong MM, Ivanov II, Min R, Victora GD (2008). TGF-beta-induced Foxp3 inhibits T(H)17 cell differentiation by antagonizing RORgammat function. Nature..

[45] Glatigny S, Duhen R, Oukka M, Bettelli E (2011). Cutting edge: loss of alpha4 integrin expression differentially affects the homing of Th1 and Th17 cells. J Immunol.

[46] Ren J, Li B (2017). The functional stability of FOXP3 and RORgammat in Treg and Th17 and their therapeutic applications. Adv Protein Chem Struct Biol.

[47] Komatsu N, Okamoto K, Sawa S, Nakashima T, Oh-hora M, Kodama T (2014). Pathogenic conversion of Foxp3+ T cells into TH17 cells in autoimmune arthritis. Nat Med.

[48] Kluger MA, Meyer MC, Nosko A, Goerke B, Luig M, Wegscheid C (2016). RORgammat(+)Foxp3(+) cells are an independent bifunctional regulatory T cell lineage and mediate crescentic GN. J Am Soc Nephrol.

[49] Hovhannisyan Z, Treatman J, Littman DR, Mayer L (2011). Characterization of interleukin-17-producing regulatory T cells in inflamed intestinal mucosa from patients with inflammatory bowel diseases. Gastroenterology..

[50] Wang T, Sun X, Zhao J, Zhang J, Zhu H, Li C (2015). Regulatory T cells in rheumatoid arthritis showed increased plasticity toward Th17 but retained suppressive function in peripheral blood. Ann Rheum Dis.

[51] Kraczyk B, Remus R, Hardt C (2014). CD49d Treg cells with high suppressive capacity are remarkably less efficient on activated CD45RA- than on naive CD45RA+ Teff cells. Cell Physiol Biochem.

[52] Issekutz AC, Issekutz TB (1995). Monocyte migration to arthritis in the rat utilizes both CD11/CD18 and very late activation antigen 4 integrin mechanisms. J Exp Med.

[53] Yednock TA, Cannon C, Fritz LC, Sanchez-Madrid F, Steinman L, Karin N (1992). Prevention of experimental autoimmune encephalomyelitis by antibodies against alpha 4 beta 1 integrin. Nature..

[54] Escribese MM, Conde E, Martin A, Saenz-Morales D, Sancho D (2007). Perez de Lema G, et al. therapeutic effect of all-trans-retinoic acid (at-RA) on an autoimmune nephritis experimental model: role of the VLA-4 integrin. BMC Nephrol.

[55] Nurieva R, Yang XO, Martinez G, Zhang Y, Panopoulos AD, Ma L (2007). Essential autocrine regulation by IL-21 in the generation of inflammatory T cells. Nature..

[56] Yang XO, Nurieva R, Martinez GJ, Kang HS, Chung Y, Pappu BP (2008). Molecular antagonism and plasticity of regulatory and inflammatory T cell programs. Immunity..

[57] Korn T, Bettelli E, Oukka M, Kuchroo VK (2009). IL-17 and Th17 cells. Annu Rev Immunol.

[58] Braza F, Brouard S, Chadban S, Goldstein DR (2016). Role of TLRs and DAMPs in allograft inflammation and transplant outcomes. Nat Rev Nephrol..

[59] Colonna L, Lood C, Elkon KB (2014). Beyond apoptosis in lupus. Curr Opin Rheumatol.

[60] Kato H, Perl A (2018). Blockade of Treg cell differentiation and function by the interleukin-21-mechanistic target of rapamycin axis via suppression of autophagy in patients with systemic lupus erythematosus. Arthritis Rheumatol..

[61] Kato H, Perl A (2014). Mechanistic target of rapamycin complex 1 expands Th17 and IL-4+ CD4-CD8- double-negative T cells and contracts regulatory T cells in systemic lupus erythematosus. J Immunol.

[62] Dolff S, Abdulahad WH, Westra J (2011). Doornbos-van der Meer B, Limburg PC, Kallenberg CG, et al. increase in IL-21 producing T-cells in patients with systemic lupus erythematosus. Arthritis Res Ther.

[63] Duarte JH, Zelenay S, Bergman ML, Martins AC, Demengeot J (2009). Natural Treg cells spontaneously differentiate into pathogenic helper cells in lymphopenic conditions. Eur J Immunol.

[64] Humrich JY, Morbach H, Undeutsch R, Enghard P, Rosenberger S, Weigert O (2010). Homeostatic imbalance of regulatory and effector T cells due to IL-2 deprivation amplifies murine lupus. Proc Natl Acad Sci U S A.

[65] Boyman O, Sprent J (2012). The role of interleukin-2 during homeostasis and activation of the immune system. Nat Rev Immunol..

[66] He J, Zhang X, Wei Y, Sun X, Chen Y, Deng J (2016). Low-dose interleukin-2 treatment selectively modulates CD4(+) T cell subsets in patients with systemic lupus erythematosus. Nat Med.

[67] Dall'Era M, Pauli ML, Remedios K, Taravati K, Sandova PM, Putnam AL (2019). Adoptive Treg cell therapy in a patient with systemic lupus erythematosus. Arthritis Rheumatol.

